# Epidemiology of child psychopathology: major milestones

**DOI:** 10.1007/s00787-015-0681-9

**Published:** 2015-02-22

**Authors:** Frank C. Verhulst, Henning Tiemeier

**Affiliations:** 1 Department of Child and Adolescent Psychiatry, Erasmus Medical Center-Sophia Children’s Hospital, P.O. Box 2060, 3000 CB Rotterdam, The Netherlands; 2 Department of Epidemiology, Erasmus Medical Center, Rotterdam, The Netherlands

**Keywords:** Epidemiology, Child psychiatry, Methodology, Longitudinal, Cohort, Prenatal

## Abstract

Child psychiatric epidemiology has developed rapidly from descriptive, cross-sectional studies in the 1960s to the current large-scale prospective cohorts that unravel aetiological mechanisms. The objective of the study was to give an overview of epidemiological studies that have influenced child psychiatry. A chronological overview of selected major milestone studies was obtained to demonstrate the development of child psychiatric epidemiology, with a more in-depth discussion of findings and methodological issues exemplified in one cohort, the Generation R Study. Epidemiological studies have been successful in describing the frequency and course of child psychiatric problems. The high expectations that biological factors can be used to better explain, diagnose or predict child psychiatric problems have not been met. More ambitious large-scale child psychiatric cohort studies are needed, carefully applying genetics, neuroscience or other molecular research to better understand how the brain produces maladaptive behaviour. Progress will only be attained if the basic sciences are systematically integrated in cohorts with rigorous epidemiological designs rather than hurriedly inserted in child psychiatric studies.

**Electronic supplementary material:**

The online version of this article (doi:10.1007/s00787-015-0681-9) contains supplementary material, which is available to authorized users.

## Introduction

Ever since the influential work of Lapouse and Monk [1], the first true child psychiatric epidemiological study published in 1958, epidemiological strategies are inseparably intertwined with research and clinical practice in child and adolescent psychiatry. Although psychiatric epidemiology is often associated with the study of the frequency of disorders in the general population, epidemiological approaches have been used to answer questions pertaining to aetiology, diagnostic assessment, treatment and prevention of child psychopathology. Epidemiological approaches to study child psychopathology have developed rapidly from descriptive, cross-sectional studies in the 1960s, often using ad hoc procedures to assess problem behaviours reported by one informant (e.g. parents or teachers), to the current large-scale prospective cohorts integrating biological measures and multi-informant behavioural assessments to unravel aetiological mechanisms.

We will present (1) a brief and selective overview of landmark epidemiological general population-based studies that have influenced child psychiatry; (2) a more detailed overview of one currently conducted large-scale longitudinal cohort study that started pre-birth, the Generation R Study; (3) a discussion of methodological issues relevant to longitudinal epidemiological research and (4) suggestions for future studies that may move child psychiatry further.

## Major milestone population-based epidemiological studies

We will present a chronological overview of selected major milestone population-based studies that mark stages in the development of child psychiatric epidemiology. However, we do not intend to give a systematic and complete overview. A number of systematic overviews of child psychiatric epidemiological studies have recently been published [2, 3] and our decision on which information to include was based on our knowledge of studies that stand out in the literature.

Table 1 gives an overview of selected milestone population-based studies. Most of these studies are longitudinal; a few cross-sectional studies will be presented because of their relevance to the field.

**Table 1:** Milestone epidemiological studies

<1960	1960–1970	1970–1980	1980–1990	1990–2000	>2000
National Survey of Health and Behavior (1946)	Isle of Wight Study (1964)	The Dunedin Multidisciplinary Health and Development Study (1972)	Montreal Longitudinal and Experimental Study (1984)	ALSPAC (1991)	GenerationR (2002)
National Child Development Survey (1958)	Sudan Study (1964/1965)	The Christchurch Health and Development Study (1977)	Pittsburgh Youth Study (1987)	NICHD (1991)	MoBa/ABC (1999, 2002)
Kauai Longitudinal Study (1955)		ASEBA Studies (1976)	Population-based twin samples	Great Smokey Mountains Study (1993)	

### Studies initiated prior to 1960

The study by Lapouse and Monk [1] was the first to systematically examine the frequency of parent-reported problems in a representative sample of 482 children aged 6–12 years from the general population. Prior to this study, the prevalence of child problem behaviours was studied only in quasi-epidemiological studies of non-representative samples. The high rates of fears and worries observed casted doubt on the, at that time, widely held opinion that these problems indicate disturbance.

Prior to 1960, two British National Birth Cohort studies were launched, one in 1946 (National Survey of Health and Development) [4] and the other in 1958 (National Child Development Survey) [5], both consisting of all births in England, Scotland and Wales in 1 week, and with an emphasis on physical health and educational and social development. These studies excelled in size and representativeness, but the assessment of behavioural problems was limited and employed poorly validated definitions of psychopathology. Yet, these studies have been very important in showing that children who, as adults, developed schizophrenia differed from their peers in terms of childhood motor, cognitive, behavioural and social characteristics [6]. This finding was confirmed by later birth cohort studies [7].

Another seminal study in the pre-1960 era is the Kauai Longitudinal Study by Werner [8, 9]. This study monitored the impact of perinatal, psychological and social risk factors in a cohort of 698 individuals, who were born in 1955 on the Hawaiian Island of Kauai, from the perinatal period to age 40 years. The authors observed how newborns with minor degrees of brain damage developed in the longer term. With the exception of serious central nervous system damage, often leading to severe mental retardation or institutionalization, the impact of perinatal complications on later adaptation diminished with time. Strikingly, the outcomes depended on the quality of the child-rearing environment. This study was one of the first to demonstrate a “washout” of the effects of early biological insults over time and of the importance of the quality of the postnatal environment interacting with these biological risks.

### Studies initiated between 1960 and 1970

A landmark in the history of child psychiatric epidemiology is the Isle of Wight study [10]. This study started in 1964 and was novel because of its two-stage sampling procedure with multi-informant and multi-method behavioural standardized assessments. Using a population-based approach, all 9-/10-year-olds on the Isle of White were screened using parent and teacher ratings, followed by intensive assessments of screen-positive and randomly selected children. Findings that stand out were: the 6.8 % prevalence of overall psychiatric disorder, the low correlation between parent and teacher ratings of child problem behaviours and the finding that most children with psychiatric disorder did not receive treatment. Comparisons with other studies [3] led to the sobering conclusion that prevalence estimates strongly differ due to methodological variation and sparked initiatives for greater standardization. Follow-up studies of this population 4 or 31 years later have been less influential [11, 12]. Few determinants had been assessed and the two-stage sampling limits power in those free of disorders at baseline.

Cederblad and co-workers utilized a quasi-experimental nature of two cross-sectional studies carried out 15 years apart in Khartoum, Sudan [13–15]. This “culturally sensitive research” [16] revealed a historical trend in the prevalence of child problem behaviours. In 1964–1965 the authors observed few problems in children living in three villages lying on the outskirts of Khartoum, Sudan, despite poor nutrition and physical health. The largely rural communities subsequently underwent rapid urbanization and economic growth resulting in better housing, nutrition, sanitation, medical care and education. Despite these improvements, the authors, who copied the methodology of the first study on to the second study, found that mothers reported more problems in their children in 1980. The authors argued that cultural factors might have accounted for this increase. For example, hyperactive behaviour was regarded as a problem if children are expected to sit still in school, but went unnoticed in the traditional rural life when far less children attended school. Currently, there is growing recognition of the need for epidemiological research addressing adaptive and maladaptive functioning across cultures [17].

### Studies initiated between 1970 and 1980

The period from 1970 to 1980 marks the start of two highly influential longitudinal epidemiological studies, both from New Zealand: the Dunedin Multidisciplinary Health and Development Study [18] and the Christchurch Health and Development Study [19, 20].

The Dunedin Study was initially driven by the question how well children with perinatal problems developed as they passed infancy. This study turned out to become one of the most influential longitudinal studies tackling a host of major developmental questions. The relatively small sample size and the restriction of the sample to one locality (1,139 eligible 3-year-old children from a 1-year cohort of children born in one hospital) were offset by the remarkable retention rate (at age 38 years, 96 % were followed up). The study showed that observed behaviour at very young age was predictive of adult psychopathology [21]. Another innovative line of research showed that childhood onset versus adolescent onset antisocial behaviours were differentially associated with risk factors and outcomes [22, 23]. Other important studies showed that adolescent cannabis use was predictive of psychotic symptoms [24].

Certainly, the most widely cited publications from this cohort are a series of studies suggesting interactions between candidate gene variations and environmental risk factors (cGxE). For example, the authors showed how a polymorphism in the MAOA gene moderates the effect of childhood maltreatment on antisocial behaviour [25], or how a polymorphism in the 5-HTT gene moderates the association between stressful life events on depression [26]. These studies have prompted many researchers to try and replicate their findings with varying success [27].

The Christchurch Health and Development Study started in 1977 with a birth cohort of 1,265 children born in Christchurch, New Zealand, with the latest follow-up at age 30 [19, 28]. Among the many results, the study increased awareness of the long-term effects of child sexual abuse and of risk factors associated with the onset of suicidal behaviour during adolescence [29, 30]. Apart from these study themes, Fergusson published many methodological aspects of longitudinal studies, especially those pertaining to measurement.

In 1976, Achenbach and colleagues sampled 1,442 children aged 4–16 years randomly from Washington D.C., Maryland, and Northern Virginia. Child problems were assessed with the CBCL [31]. This generated normative data for child problem behaviours as reported by parents. Achenbach subjected scores for specific problem items to factor analysis to form scales and evaluated the scale scores in relation to population norms [32]. This empirical approach to assessing child problems, competencies and adaptive functioning was termed the Achenbach System of Empirically Based Assessment (ASEBA), of which the widely used Child Behavior Checklist (CBCL) is the prototype. The ASEBA forms span a wide age range making this approach valuable for longitudinal epidemiological studies. This approach was extended to assessments by other informants and methods, and for different ages and cultures, thus building a framework of multi-informant, multi-method and multicultural assessment that is used in numerous studies all over the world [32].

### Studies initiated between 1980 and 1990

From this period, the Montreal Longitudinal and Experimental Study [33], launched in 1984, and the Pittsburgh Youth Study [34], launched in 1987, stand out. The Montreal Longitudinal and Experimental Study consisted of 1,034 Canadian boys of low socioeconomic status who were assessed in kindergarten by their classroom teacher and annually by their teacher between ages 10 and 15 years [33, 35]. Self-reported delinquency measures were obtained after puberty. The main aim was to study early behavioural predictors of later antisocial behaviours. Groups of children were identified who follow distinctive developmental trajectories with a technique novel at that time to child psychiatric epidemiology. Distinct trajectories were found to have different outcomes. For example, boys following a chronic physical aggression trajectory were likely to show physical violence and be involved in the most serious delinquent acts [35].

The Pittsburgh Youth Study [34] is a longitudinal study of male antisocial behaviour. A random sample of inner-city Pittsburgh boys attending first, fourth and seventh grades in public schools was assessed. To this group, the authors added boys with the most severe disruptive behaviour problems. The resulting high-risk sample of 1,517 boys was followed for 21 years with (for the youngest cohort) 19 assessment waves. A sad indication of the success of this sampling strategy was that during follow-up, 37 boys were convicted for committing homicide and another 39 were homicide victims. The authors found evidence for a gradual developmental progression from less serious to serious antisocial behaviours along three pathways identified as overt, covert and authority conflict pathways. The authors were able to predict who became a serious offender (risk factors include poverty, living in a disadvantaged neighbourhood, poor child-rearing practices), but not which serious offenders will desist. Important conclusions of their work are that almost all homicide offenders had committed violent acts before committing homicide, almost all homicides were “street” homicides rather than homicide of relatives, and, interestingly, the majority of homicide offenders did not have a psychopathic personality.

Twin studies showed that genetic factors explained a significant proportion of individual variation in psychopathology. Because a heritability estimate is a population-specific snapshot, and most heritability estimates were based on samples of convenience [36], there emerged a need for representative epidemiological twin samples. Large general population-based twin registries were formed, including the Virginia Twin Registry (started in 1987) [37, 38], the Netherlands Twin Registry (started in 1987) [39] and the Swedish Twin Study of Child and Adolescent Development (TCHAD; started in 1993) [40]. Across these population-based twin samples, heritability estimates for most common child psychiatric disorders were derived. Later research using longitudinal twin data added information on the influence of genetic (and environmental) stability on the stability of child psychopathology [39]. Further, twin studies now incorporate more biological and environmental measures to also conduct classical risk factor epidemiology.

### Studies initiated between 1990 and 2000

The prototypical prospective study designed to include child behavioural and cognitive but also biological measures is the Avon Longitudinal Study of Parents and Children (ALSPAC) [41]. This birth cohort enrolled over 14,000 mothers during pregnancy in 1991 and 1992. ALSPAC, to this very moment, has been a very productive study with numerous highlights. One focus of the behavioural studies embedded in the ALSPAC cohort was the effect of nutrition during pregnancy on neurodevelopment. Both low maternal fish intake and low maternal iodine urine concentrations during pregnancy were associated with poor child cognitive outcomes [42]. Another series of studies from ALSPAC related maternal depression and anxiety during pregnancy to child behavioural problems [43]. Perhaps, the methodological work embedded in the ALSPAC which addressed confounding made the most impact on child psychiatry.

The Great Smokey Mountains Study was launched in 1993 [44]. Children between 9 and 13 years who were screened positive for externalizing problems were oversampled, as were American Indian children. This survey of child psychiatric problems became a fruitful longitudinal study. It found that the sharp increase in depression in girls was associated with pubertal stage transition and this was entirely explained by increasing levels of sex hormones [45]. A classical finding stems from a natural experiment in which an entire community of American Indians experienced significant income increases as a result of the opening of a gambling casino. Many families moved out of poverty, and the originally higher externalizing, but not the internalizing problems of the children decreased to levels similar to those of children who had never been poor [46]. These results supported a social causation explanation for conduct and oppositional behaviours, but not for anxiety or depression.

The *NICHD* Study of Early Child Care and Youth Development is a longitudinal study to answer questions about the relationships between childcare and children’s developmental outcomes [47]. The study started in 1991 with a sample of 1,364 children followed from 1 month of age through grade 9 when children were about 15 years old. A highly debated conclusion was that early and extensive non-maternal care increased the probability of insecure infant–parent attachment relationships, child aggression and noncompliance during the toddler, preschool and early primary school years [48]. The effects were not attributed to low-quality care, nor did they merely reflect assertiveness rather than true aggression. However, the negative effects of early non-maternal childcare were modest and did not predict clinical levels of later child psychopathology [49]. Other authors used the publicly available NICHD data to address the impact of maternal sensitivity or harsh parenting on child behaviour showing that better quality parenting predicts fewer problems and poorer quality parenting predicts more problems. Using this cohort’s data, Belsky [50] developed his differential susceptibility model suggesting that persons vary in their susceptibility to adverse and enriching experiences.

### Studies initiated from 2000 onwards

More recently, a number of birth cohorts were started, all including data collection in the antenatal period [2], but only two had a clear focus on child behavioural and cognitive development: (1) the Norwegian Mother and Child Cohort Study (MoBa) [51] and (2) the Dutch Generation R Study [52, 53], which will be discussed separately.

The MoBa study includes 109,000 children born from 1999 to 2009. Mothers were recruited at ultrasound examinations around week 18 of gestation. Parent questionnaires, referral information and linkages to the Norwegian Patient Registry identified cases of ASD, ADHD, epilepsy and cerebral palsy in the cohort. The strength of this study is its large sample size, which makes is suitable for the study of infrequent disorders such as autism. In one study it was found that the use of prenatal folic acid supplements around the time of conception was associated with a lower risk of autistic disorder [54]. The investigators also linked autism to reduced head growth patterns [55].

## The Generation R Study

The Generation R Study (“R” for Rotterdam) is a population-based cohort of children who are followed from foetal life forwards. This multi-ethnic cohort consists of 9,778 pregnant women and their live-born children. A subgroup of 1,106 women and their children received in-depth assessments. The overall aim of the study was to identify early determinants of children’s growth, development and health, including behavioural, cognitive and social development. A specific aim was to test the foetal origins hypothesis stating that disproportionate foetal growth programs later disease [56].

Among its many measures, we believe that Generation R, compared to other cohort studies, stands out for the following: (1) the use of three prenatal ultrasound measures of the foetal head, brain (including ventricles) and other body parts, enabling the computation of prenatal growth curves; (2) its observational measures, including observations of the home environment, parent–child attachment relationship, parental sensitivity, the child’s compliance, temperament, emotion recognition and cheating; (3) neurodevelopmental measures, including postnatal 3D brain ultrasound, motor assessment, executive functions, IQ and language, and brain MRI (structural, DTI and resting state fMRI) and (4) reports on child problem behaviours by multiple informants: fathers, mothers, teachers, child self-reports and peer nominations [52, 53].

We highlight three research themes that, to our opinion, have generated new insights: (1) associations between maternal prenatal exposures and foetal growth; (2) associations between foetal growth and postnatal child development and (3) associations between social disadvantage and foetal growth (see, supplementary Table 2 for an overview of major findings, recommendations and methodological challenges of the prenatal and early childhood neurodevelopmental and behavioural studies in Generation R; supplementary Table 3 gives an overview of references of published prenatal and early childhood neurodevelopmental and behavioural studies in Generation R).

### Prenatal exposures and foetal growth

The repeated prenatal ultrasounds made it possible to study the impact of maternal exposures on trajectories of foetal head growth, which reflects early neurodevelopment. Similarly, indictors of foetal cerebral blood flow have been assessed by ultrasound to study neurodevelopment. Maternal smoking, cannabis or SSRI use, low folate during pregnancy and prenatal maternal depression, anxiety and family stress were all associated with less foetal head growth, with relatively strong effect estimates observed for maternal smoking, SSRI and cannabis use [57–59]. A finding that stood out was the substantial negative effect of (subclinical) hypothyroidism on foetal growth and birth outcomes [60]. We concluded that numerous intrauterine exposures are related to foetal growth from the first to third trimester.

### Foetal growth and postnatal child development

Low birth weight is associated with several psychiatric problems, including depression and hyperactivity, and poor cognitive functioning [61, 62]. However, birth weight is an end point measure of foetal growth and does not inform us about variations in intrauterine growth. Therefore, we tested whether prenatal exposures impacted on preschool child behaviour and cognition and to what extent this was mediated by variations in foetal growth trajectories. Although we found that intrauterine head growth was associated with delayed early motor development, there was little indication that intrauterine growth was associated with problem behaviour, temperamental difficulties or cognition [63, 64]. Also, we found that maternal exposures negatively affecting intrauterine growth, such as exposures to nicotine or cannabis, had little, if any, impact on later child problem behaviour if carefully controlled for confounding [63]. If prenatal environmental exposures were associated with later problem behaviours in the preschool period, as was the case for family stress or parental psychopathology, there was no mediation by foetal growth, suggesting that much of the observed intrauterine association was due to genetic confounding or spill over of parental behaviour from the prenatal to the postnatal period [65]. In conclusion, these findings support the view that several negative effects of prenatal environmental exposures can be compensated later in life. Hence, detailed observations of foetal growth in the Generation R cohort did not support the Barker hypothesis. Of course, we do not know yet what the impact of these environmental exposures is on later functioning at school age and in adolescence.

### Social disadvantage and foetal growth

The impact of social disadvantage can be detected from childhood onwards. We found compelling evidence that socioeconomic differences can already be found in the intrauterine period and that this extends into the child’s postnatal development. Low maternal education was not only associated with premature birth and low birth weight, but also with less foetal growth. Importantly, social differences were detectable in foetal brain growth more than in that of peripheral and abdominal tissues [66]. The association between poor foetal growth and child problem behaviours could largely be explained by parental psychosocial and maternal lifestyle characteristics [63]. Socioeconomic inequalities could also be documented for early temperamental traits and problem behaviour in young children [67]. This indicates that health inequalities accumulate in disadvantaged families from the intrauterine period of children’s life onwards.

So far, most Generation R studies have shown that individual differences arise from a large number of causal factors, with each contributing a relatively small effect. This cumulative risk model suggests that the additive contribution of genetic, perinatal and environmental risks puts the child at a progressively greater risk, despite the small impact that any single factor is likely to have.

## Methodological considerations

### Confounding and causal inference

Associations observed in prospective studies may help to infer causality because of the temporal relationship between risk factor and outcome and the possibility to study within-individual change. However, there are a number of caveats. One type of confusion may arise from the distortion of the effect of an exposure of interest by some extraneous factor when the effect of the extraneous factor is mistaken for or mixed with the actual exposure effect. This is the well-known problem of confounding. To illustrate, think of the association between smoking by the mother during pregnancy and child problem behaviour. This association disappeared in the Generation R Study after controlling for educational level, national origin and maternal psychopathology [68]. This type of statistical approach relies on adequate measurement of confounding factors, but cannot deal with residual confounding caused by unmeasured or unrecognized confounding factors. This is the case with heritable characteristics or behaviours that are commonly overlooked as potential residual confounders. For instance, environmental studies of child IQ often cannot control for maternal or paternal IQ as this was not assessed. Studies of intrauterine influences (e.g. smoking) may reflect unrecognized heritable maternal behaviour. Of course, mothers transmit genes to their children and it may therefore be possible that associations between prenatal exposure and child outcomes arise from genetic variations mothers and children share and not from a direct effect of prenatal exposure.

Several epidemiological approaches have been developed to help tackle this important source of bias. Increasingly, they are also applied in child psychiatry. One approach to address residual confounding is studying two or more siblings from each family, which allows testing environmental exposures such as exposure to intrauterine smoking [69]. The sibling design automatically rules out environmental differences that vary between families, such as ethnicity, socioeconomic status or neighbourhoods. It can overcome genetic confounding as parents randomly distribute genetic variants among siblings. The main limitations of the approach are the availability of differentially exposed siblings and the validity of changing exposure information.

An indication of residual confounding in studies of intrauterine exposure can also be obtained by comparing maternal–paternal exposure associations. This maternal–paternal approach compares the magnitude of the association of the maternal exposure with child outcome to that of the equivalent association for paternal exposure with child outcome. A biological intrauterine effect would be expected to produce a stronger maternal association, compared with the paternal association, since paternal exposures would not normally be expected to affect the intrauterine environment. For example, there were no differences in associations of prenatal maternal and paternal overweight with child problem behaviour. Hence, there was little evidence for a causal intrauterine effect of maternal overweight during pregnancy [70].

Another approach recommended for many years to address causality, Mendelian randomization, has rarely been applied in studies of intrauterine influences in child psychiatric epidemiology. Mendelian randomization is a method that uses variation in genes of known function to examine the causal effect of a risk factor on disease. Only a few examples exist where a known relation between a genetic variant and an exposure, here intrauterine exposure to alcohol, could be used as a causal effect of alcohol on cognition. The alcohol dehydrogenase gene (ADH1B) has been studied in the ALSPAC to demonstrate that maternal alcohol use during pregnancy most likely underlies effects on educational achievement [71]. In general, modifiable exposures in child psychiatric epidemiology have no known genetic determinants precluding Mendelian randomization to demonstrate causality.

### Reverse causality

One way to understand the causal pathway between an exposure and outcome is to determine mediation mechanisms. This approach starts with a general factor (e.g. social disadvantage) and then tries to determine whether the causal inference can be accounted for by more specific elements in this broader variable (e.g. parenting style, physical home environment). Many studies have shown that social disadvantage is associated with young children’s problem behaviours. However, less is known about the mechanisms underlying this association. In the Generation R Study, we found that the association between social disadvantage and child problem behaviours was mediated by maternal emotional well-being, disrupted parenting and characteristics of the observed physical home environment such as the availability of learning materials [72]. From these findings, we may conclude that maternal depression or parental stress causes child problem behaviour, but the reverse may also be true, namely that the child’s problems elicit parental stress.

Cross-sectional studies are notorious for their inability to determine the direction of association. Longitudinal studies, in which the exposure predates the outcome, are much better suited to infer how risk factors impact psychopathology that occurs during follow-up. But even if it is possible to control well for baseline levels of psychopathology, longitudinal studies can hardly ever rule out reverse causality. In particular in psychiatry, with its varying levels of symptom severity across time, reverse causality can arise if subclinical problems or the history of psychopathology is not controlled for. Cohort studies starting in the intrauterine period are better set to exclude reverse causality in studies of child outcomes, as some parental factors can be measured before the child is born. Yet, few birth cohorts have investigated whether negative effects of the behaviour of the child extend to the family system, such as parental psychopathology or family functioning. Mostly, such studies were performed controlling for child behaviour at baseline, but often the available measures do not allow ruling out reciprocal associations very well. However, examining the bi-directionality of relations between the child and his or her environment is key to understanding the persistence of problem behaviour.

### Shared method variance bias

A common problem in studies on child development is shared method variance, which is the variance that is attributable to the measurement method or informant reports rather than to the constructs the measures are assumed to represent. For example, if mothers report on their own emotional problems and on their child’s behaviour, which is common in many large-scale studies in very young children, there is the likelihood of halo effects in ratings, reflecting shared method variance. This is particularly important if maternal traits such as hostility are studied, since hostility may influence the perception of child problems [73].

Shared method variance bias can be reduced by using multiple sources of information (e.g. mothers’, fathers’, teachers’ reports, children’s self-reports and peer nominations) and multiple methods (e.g. interviews, questionnaires, observational assessments or tests). In Generation R, most studies used a multi-method, multi-informant approach enabling us to test associations with different assessment methods or informants for the determinants versus outcomes, thereby reducing error due to common-method variance. The use of multiple information sources makes it possible to test the consistency of findings. For example, in Generation R, we found that maternal and paternal self-reported depression was equally associated with problems as reported by children themselves. However, if mothers reported on her child’s problems, maternal depression had a much stronger association than paternal depression with child problems; likewise, paternal depression had a stronger association with child problems if reported by the father. This strongly suggests that if parents report both on their own and their child’s problems, associations tend to be inflated [74].

### Replication

Not only the discovery, but also the replication of associations is a core activity of aetiological research. Ioannidis [75] argued that discovery could be exploratory, opportunistic and perhaps even selective if replication attempts are frequent. For replication studies, a more restrained approach is desirable, in particular exposure and outcome must be similar to the discovery study. Also, the age range and the follow-up duration should be comparable; else, replication efforts become a premature test of the generalizability of not replicated results. Replication can test the biases that may affect the initial study. Large cohort studies offer ideal settings for discovery and replication efforts; they allow a large discovery sample and similar assessments are available for immediate replication. Yet, the discovery study in child psychiatry is rarely accompanied by an immediate replication effort. Most interesting exploratory analyses are pushed forward in stand-alone publications. Recently, and driven by the critique on the initial gene–environment interaction studies, this practice is changing. The early findings of genetic susceptibility to stress and maltreatment from the Dunedin cohort were published in science, solely presenting discovery analyses. Five years later, the initial evidence from the Dunedin cohort that effects of breastfeeding on IQ may partly depend on a single genetic variant included a successful replication in a British birth cohort [76]. Interestingly, neither approach yielded findings that have since been replicated conclusively.

### Selection bias

Selection bias in cohort studies can occur at baseline because of refusal to participate and later due to loss to follow-up. Poor baseline response can affect generalizability and validity, although a much-cited publication showed that even for a baseline response as low as 30 % the effect of non-participation was small [77]. Generally, cohort researchers worry more about the second phenomenon, loss to follow-up. The Dunedin Study achieved remarkable follow-up rates exceeding 90 % at most waves; more recent large-scale studies such as the Danish National Birth Cohort could retain only 60 % of families included at baseline [78]. The national health registry in Denmark with excellent coverage enabled the investigators to demonstrate that the presence and magnitude of bias due to this modest follow-up rate may be less than feared. Further, bias strongly depended on the exposure and outcome under study. However, child psychiatric epidemiologists cannot relax: the most pronounced bias was found in the association of maternal smoking and ADHD [79]. Similarly, researchers in ALSPAC concluded that follow-up rates only marginally affected longitudinal associations, although the observed associations between maternal smoking and behaviour differed by more than 30 % between responders and non-responders [80].

## Suggestions for future studies

Epidemiological studies have been successful in describing the frequency and course of child psychiatric problems. Aetiological research clearly demonstrated the strong associations between social disadvantage and child maltreatment with behavioural and emotional development. In contrast, the high expectations that biological factors can be used to better explain, diagnose or predict child psychiatric problems have not been met.

Future risk factor epidemiology in child psychiatry will undoubtedly follow the lead of cardiovascular and cancer research. After the hype of candidate gene, cGXE and GWAS studies, child psychiatrists are already focussing on epigenetic, microbiomic and metabolomic studies. Yet, child psychiatry has hardly harvested the fruit on the GWAS tree. So far, published studies of common child psychiatric problems have been hopelessly underpowered. If schizophrenia researchers need to include more than 30,000 affected individuals to find several genetic loci, child psychiatrists must certainly include equal numbers of children with disorders or, because large mono-site samples may not be feasible, pool cohort studies exceeding 100,000 children to find associations between genetic variants and common psychiatric disorders or traits. This is not so much because child psychiatric disorders are less heritable, but mainly because disorders are less specific, not precisely measured and subject to developmental changes. Pooling across multiple sites, either within or across nationalities, should ideally comprise information on individuals that were sampled and assessed according to the same protocol as was done, for example, in the IMAGEN study [81]. Pooling of samples from ongoing studies that use different methodologies has the disadvantage that only the limited number of confounders, which were measured uniformly across studies, can be taken into account. Of course, natural experiments, such as the Romanian adoption studies [82], or studies using high-risk samples, such as the Mannheim Study of Children at Risk [83] can also be useful to study the aetiology of child psychopathology, but fall outside the scope of this selected review.

The future epigenetic, metabolomics and microbiomic studies should adhere to the lessons learnt from the (non-psychiatric) GWAS studies. The scientific rigor with multiple hypothesis testing and immediate replication must be adapted and applied before child psychiatrists embrace this new research opportunity. In the meantime, child psychiatry may devote some attention to new environmental risks provided by extensive media use and chemicals. Phthalates and bisphosphonates are toxins with a presumably neurodevelopmental effect. If not cohort researchers, who is in the position to assess the impact of these compounds?.

Traditionally, child psychiatric researchers have relied on continuous phenotype measures, both in clinical practice and research. Likewise, child psychiatrists have recommended a multiple informant approach to assess child psychopathology. Although implemented in clinical practice, researchers do not systematically adhere to this advice. In particular in biological studies such as neuroimaging or genetic research, few examples of multiple informant assessments can be found. Future studies can improve precision and validity of results by complementing mother reports with father, child self, peer or teacher reports of child problem behaviours.

Clinical cohorts have successfully incorporated neuroimaging techniques in both cross-sectional and longitudinal design. Notably, a maturational delay was demonstrated in children with ADHD and abnormal neurodevelopmental processes in adolescents with early-onset schizophrenia [84, 85]. These findings cannot unravel the direction of the association between disorder and brain development. Population neuroscience is needed to demonstrate whether brain alterations precede the onset of psychiatric problems. This implies that large-scale imaging studies in very young children from the general population are performed.

In summary, future research in child psychiatry will most probably continue to employ sophisticated epidemiological approaches. Multiple imputations, immediate replication, models of causal inference, repeated measures and multiple informants are increasingly standard practice. Yet to apply genetics, neuroscience or other molecular research to better understand how the brain produces maladaptive behaviour, we need to conduct more ambitious large-scale child psychiatric cohorts. Progress is booked only if we integrate the basic science with more rigorous epidemiological designs.

## Five key points

- Epidemiological studies have been successful in describing the frequency and course of child psychiatric problems.
- The high expectations that biological factors can be used to better explain, diagnose or predict child psychiatric problems have not been met.
- Epidemiology has shown that individual differences in child neurodevelopment arise from a large number of causal factors, with each contributing a relatively small effect.
- Published studies of common child psychiatric problems have been hopelessly underpowered.
- To apply genetics, neuroscience or other molecular research to better understand how the brain produces maladaptive behaviour, we need to conduct more ambitious large-scale child psychiatric cohort studies or pool studies that use similar methodologies across multiple sites.

## Electronic supplementary material

Below is the link to the electronic supplementary material.

Supplementary material 1 (DOCX 69 kb)

Supplementary material 2 (DOCX 32 kb)
