# Supplementary material for: Epidemiology of child psychopathology: major milestones
Source: Eur Child Adolesc Psychiatry. 2015 Feb 22;24(6):607–17. doi: 10.1007/s00787-015-0681-9 (PMC4452764; doi:10.1007/s00787-015-0681-9)
Supplement: Supplementary file 1 — Supplementary material 1 (DOCX 69 kb) [file 787_2015_681_MOESM1_ESM.docx]

Supplementary table 2

Overview of major findings, recommendations, and methodological challenges of the prenatal and early childhood neurodevelopmental and behavioural studies in Generation R

| **DETERMINANT** | **FINDINGS**  **ASOCIATIONS WITH CHILD OUTCOMES** | **METHODOLOGICAL CHALLENGE** | **REMARK & SUGGESTIONS** |
| --- | --- | --- | --- |
| **Genetic variations** |  |  |  |
| Candidate genes | FTO, FKBP5, 5HTT related to face recognition food responsiveness, or cortisol reactivity[[1-3](#_ENREF_1)] | Low prior probability of any genetic association | Candidate gene studies in child psychiatry should be discontinued for now, awaiting GWAS results |
| Gene-environment  interacton study | GxE Interactions reported for candidate genes DRD4, 5HTT and COMT with maternal anxiety, parenting, or smoking [[4-7](#_ENREF_4)] | Low power, low prior probability, few replications successful | Replicate all findings prior to publication; attempt GWAS based GxE studies |
| GWAS-studies | No association in GWAs meta-analysis of Internalizing problem and Aggression, genetic association for early language delay was replicated[[8](#_ENREF_8),[9](#_ENREF_9)] | Power remains insufficient even if many child psychiatric cohorts are combined | Larger meta-analyses needed; longitudinal phenotypes may further improve precision |
| Epigenetic studies | Candidate epigenetic study of 11 methylation sites and ADHD symptoms[[10](#_ENREF_10)] | Low prior probability, confounding by environmental factors | Replication studies are challenging due to lack of comparison samples |
| **PRENATAL** |  |  |  |
| **Parental Psychopathology** |  |  |  |
| Maternal psychiatric problems | Association with foetal growth, motor development, and child problem behaviour; no association with observed infant-mother attachment[[11-15](#_ENREF_11)] | Shared method variance bias; residual confounding by genetic and other familial factors; prenatal symptoms are a proxy for postnatal problems | Other informants on child behaviour needed to overcome bias: child self and teacher report |
| Paternal psychiatric problems | Associations patterns of paternal prenatal problems with child outcomes largely similar to those of maternal problems[[13](#_ENREF_13),[16](#_ENREF_16),[17](#_ENREF_17)] | No direct paternal prenatal effect on child development known | Results are suggestive of confounding patterns in maternal prenatal associations |
| **Family stress** | Associations with fetal growth, child problem behaviour and cortisol rhythm [11, 14, 18] | Family stress is closely intertwined with maternal psychiatric problems | Important risk indicator for child health |
| **Parental substance use during pregnancy** |  |  |  |
| Alcohol | No associations detected with problem behavior [19] | Few fetuses in Generation R were exposed to heavy or chronic alcohol use | Very modest drinking during pregnancy is a proxy of good maternal health |
| Cigarette smoking | Associations with head growth, no consistent association with aggressive behavior [17, 20] | The same genetic variations may underlie both maternal nicotine use and child aggression | Null finding is consistent with other studies |
| Cannabis | Associations with fetal growth and girls’ problem behavior [21,22] | To differentiate effects of prenatal cannabis exposure from effects of smoking and other risk behaviours is difficult | Prenatal cannabis exposure may have strong effects but confounding is likely and exposure is not common (3%) |
| **Medication (SSRI) use** | Associations with fetal growth and autistic traits [23,24] | Confounding by indication; Contrasting exposures such as to other medication or untreated maternal symptoms help interpret findings | Specific effects of prenatal SSRI exposure but not of maternal psychiatric symptoms on child outcomes were observed; RCTs are feasible |
| **Diet** |  |  |  |
| Nutritional patterns | Mediterranean diet associated with problem behaviour [25] | Consider residual confounding as Mediterranean diet is a non-specific proxy of healthy lifestyle | Nutritional biomarkers and Mendelian randomization may address confounding |
| Biomarkers | Folate associated with internalizing problems, no effect of vitamin B 12 and iodium on problem behavior [26,27,28] | Small effects of folate levels and supplementation | Repeated biomarker assessments per individual should be considered |
|  |  |  |  |
| **Fetal growth** |  |  |  |
| Fetal head growth | No association with temperament, autistic traits, but with motor development [29,30] | It is important to control for effects of confounders on both the foetal size and the foetal growth (using interaction terms) | Little evidence that Barker hypothesis is relevant for common child problem behaviour |
| Fetal size/ birth weight | Curvilinear association with ADHD symptoms [31] | Confounding by maternal anthropometrics (e.g., height) |  |
| **Maternal thyroid function** | Hypothyroxinemia (low FT4 and normal TSH) associated with low IQ, autistic traits and large head size [32,33,34] | TSH less informative during pregnancy, measure in early pregnancy important | Hypothyroxinemia is an important risk factor for neurodevelopment |
| **EARLY CHILDHOOD** |  |  |  |
| **Sociodemographics** |  |  |  |
| Parental socio-economic  status, education, income,  and marital status | Low SES associated with child problem behavior; social disadvantage mediated by harsh parenting and parental psychiatric problems [35,36] | Only a small fraction of observed SES effects can be explained | SES is an established risk factor that is not fully explained |
| Ethnicity | Ethnicity is risk factor for child problem behavior mediated by poor education, maternal psychopathology, low acculturation and low income [37] | Very different ethnicities with different migration backgrounds | Only large ethnic sub-cohorts and culturally sensitive assessments can help understand specific problems |
| Home environment | Physical home, learning and social environment predicts child problem behavior [38] | Associations between observational measures were mostly not independent of parent reported socio-demographic factors | Home observation can help explain and address the association between SES indicators and child problem behaviour |
| **Parental psychopathology** |  |  |  |
| Maternal psychiatric  problems | Associated with child problem behaviour and face recognition; not associated with attachment, maternal depression mediates SES effects [14,15,36,39,40] | Genetic contribution to the effect of maternal psychiatric problems cannot be addressed without a genetically sensitive design | Multiple informant and multi-method approach can address shared method variance bias |
| Paternal psychiatric problems | Associated with child problem behavior [14] | See above |  |
| **Parenting** |  |  |  |
| Sensitivity, observed | Associations with internalizing problems, poor executive functioning and face recognition [41,42,43] | Observed sensitivity is a dyadic measure with good stability over time | Key measure to understand child behaviour; integrating observational parenting measures in large cohort studies is challenging |
| Discipline, observed | Association with poor executive functioning [44] | Laboratory and situational effects may determine child behavior and reduce precision | Reporter bias in parenting measures can be overcome |
| Harsh Parenting, self reported | Strong associations with child behavioural problems [45] | Reversed causality may partly explain effects | Harsh parenting is frequent and an important predictor of child psychiatric problems |
|  |  |  |  |
| **Attachment** | Infant-mother relationship in Strange Situation is associated with compliance; not associated with executive functioning [46,47] | This highly standardized measure cannot easily be implemented and coded in large studies. | Small effects on child psychiatric problems |
| **Breatfeeding** | Not associated with IQ [48] | Confounding, in particular by maternal IQ | Small effects, if any |
| **TV-exposure** | Associated with persistence of externalizing problems but not with occurrence of problems or bullying [49,50] | Assessment of exposure duration should be coupled with measures of content and other media use | Research on media exposure remains important due to changing media habits |
| **Bullying** | Associated with low IQ, not associated with overweight [51,52] | Peer report per classroom assessed to obtain valid measure | Class mates |
| **Neuroimaging** |  |  |  |
| **Postnatal ultrasound** | Ventricle size, corpus callosum and thalamus assessed via anterior fontanel, structure size related to anxiety symptoms and inhibition [53,54,55] | Crude measure largely measured in 2 D only | Unique imaging measure to cost-effectively assess brain structures in infants |
| **Structural brain MRI imaging** | Associations with ADHD and autistic symptoms [56,57] | Curvilinear brain growth patterns makes interpretation of cross-sectional studies in pre-adolescents difficult | Specific assessment of neurodevelopment with cortical thickness, gyrification and several structural measures |
| **Rs-fMRI** | Associations with IQ [58] | Validity not clear | DTI and rs-fMRI help determine brain connectivity implicated in behavioural problems |
