# Supplementary material for: Epidemiology of child psychopathology: major milestones
Source: Eur Child Adolesc Psychiatry. 2015 Feb 22;24(6):607–17. doi: 10.1007/s00787-015-0681-9 (PMC4452764; doi:10.1007/s00787-015-0681-9)
Supplement: Supplementary file 2 — Supplementary material 2 (DOCX 32 kb) [file 787_2015_681_MOESM2_ESM.docx]

Supplementary table 3

Overview of references of published prenatal and early childhood neurodevelopmental and behavioural studies in Generation R

1. Luijk MP, Velders FP, Tharner A, van Ijzendoorn MH, Bakermans-Kranenburg MJ, et al. (2010) FKBP5 and resistant attachment predict cortisol reactivity in infants: gene-environment interaction. Psychoneuroendocrinology 35: 1454-1461.

2. Velders FP, De Wit JE, Jansen PW, Jaddoe VW, Hofman A, et al. (2012) FTO at rs9939609, food responsiveness, emotional control and symptoms of ADHD in preschool children. PLoS One 7: e49131.

3. Szekely E, Herba CM, Arp PP, Uitterlinden AG, Jaddoe VW, et al. (2011) Recognition of scared faces and the serotonin transporter gene in young children: the Generation R Study. J Child Psychol Psychiatry 52: 1279-1286.

4. Cents RA, Tiemeier H, Velders FP, Jaddoe VW, Hofman A, et al. (2012) Maternal smoking during pregnancy and child emotional problems: the relevance of maternal and child 5-HTTLPR genotype. Am J Med Genet B Neuropsychiatr Genet 159B: 289-297.

5. Kok R, Bakermans-Kranenburg MJ, van Ijzendoorn MH, Velders FP, Linting M, et al. (2013) The role of maternal stress during pregnancy, maternal discipline, and child COMT Val158Met genotype in the development of compliance. Dev Psychobiol 55: 451-464.

6. Velders FP, Dieleman G, Cents RA, Bakermans-Kranenburg MJ, Jaddoe VW, et al. (2012) Variation in the glucocorticoid receptor gene at rs41423247 moderates the effect of prenatal maternal psychological symptoms on child cortisol reactivity and behavior. Neuropsychopharmacology 37: 2541-2549.

7. Windhorst DA, Mileva-Seitz VR, Linting M, Hofman A, Jaddoe VW, et al. (2014) Differential susceptibility in a developmental perspective: DRD4 and maternal sensitivity predicting externalizing behavior. Dev Psychobiol.

8. Benke KS, Nivard MG, Velders FP, Walters RK, Pappa I, et al. (2014) A genome-wide association meta-analysis of preschool internalizing problems. J Am Acad Child Adolesc Psychiatry 53: 667-676 e667.

9. St Pourcain B, Cents RA, Whitehouse AJ, Haworth CM, Davis OS, et al. (2014) Common variation near ROBO2 is associated with expressive vocabulary in infancy. Nat Commun 5: 4831.

10. van Mil NH, Steegers-Theunissen RP, Bouwland-Both MI, Verbiest MM, Rijlaarsdam J, et al. (2014) DNA methylation profiles at birth and child ADHD symptoms. J Psychiatr Res 49: 51-59.

11. Henrichs J, Schenk JJ, Roza SJ, van den Berg MP, Schmidt HG, et al. (2010) Maternal psychological distress and fetal growth trajectories: the Generation R Study. Psychol Med 40: 633-643.

12. van Batenburg-Eddes T, de Groot L, Huizink AC, Steegers EA, Hofman A, et al. (2009) Maternal symptoms of anxiety during pregnancy affect infant neuromotor development: the generation R study. Dev Neuropsychol 34: 476-493.

13. Van Batenburg-Eddes T, Brion MJ, Henrichs J, Jaddoe VW, Hofman A, et al. (2013) Parental depressive and anxiety symptoms during pregnancy and attention problems in children: a cross-cohort consistency study. J Child Psychol Psychiatry 54: 591-600.

14. Velders FP, Dieleman G, Henrichs J, Jaddoe VW, Hofman A, et al. (2011) Prenatal and postnatal psychological symptoms of parents and family functioning: the impact on child emotional and behavioural problems. Eur Child Adolesc Psychiatry 20: 341-350.

15. Tharner A, Luijk MP, van Ijzendoorn MH, Bakermans-Kranenburg MJ, Jaddoe VW, et al. (2012) Maternal lifetime history of depression and depressive symptoms in the prenatal and early postnatal period do not predict infant-mother attachment quality in a large, population-based Dutch cohort study. Attach Hum Dev 14: 63-81.

16. Brion MJ, Zeegers M, Jaddoe V, Verhulst F, Tiemeier H, et al. (2011) Intrauterine effects of maternal prepregnancy overweight on child cognition and behavior in 2 cohorts. Pediatrics 127: e202-211.

17. Roza SJ, Verhulst FC, Jaddoe VW, Steegers EA, Mackenbach JP, et al. (2009) Maternal smoking during pregnancy and child behaviour problems: the Generation R Study. Int J Epidemiol 38: 680-689.

18. Saridjan NS, Huizink AC, Koetsier JA, Jaddoe VW, Mackenbach JP, Hofman A, Kirschbaum C, Verhulst FC, Tiemeier H. Do social disadvantage and early family adversity affect the diurnal cortisol rhythm in infants? The Generation R Study. Horm Behav. 2010:57:247-54.

19. Bakker R, Pluimgraaff LE, Steegers EA, Raat H, Tiemeier H, Hofman A, Jaddoe VW. Associations of light and moderate maternal alcohol consumption with fetal growth characteristics in different periods of pregnancy: the Generation R Study. Int J Epidemiol. 2010;39:777-89.

20. Roza SJ, Verburg BO, Jaddoe VW, Hofman A, Mackenbach JP, Steegers EA, Witteman JC, Verhulst FC, Tiemeier H. Effects of maternal smoking in pregnancy on prenatal brain development. The Generation R Study. Eur J Neurosci. 2007; 25:611-7

21. El Marroun H, Tiemeier H, Steegers EA, Jaddoe VW, Hofman A, Verhulst FC, van den Brink W, Huizink AC. Intrauterine Cannabis Exposure Affects Fetal Growth Trajectories: The Generation R Study. J Am Acad Child Adolesc Psychiatry 2009 Dec;48(12):1173-81

22. El Marroun H, Hudziak JJ, Tiemeier H, Creemers H, Steegers EA, Jaddoe VW, Hofman A, Verhulst FC, van den Brink W, Huizink AC. Intrauterine Cannabis Exposure Leads to More Aggressive Behavior and Attention Problems in 18-Month-Old Girls. Drug Alcohol Depend. 2011;118(2-3):470-4.

23. El Marroun H, Jaddoe VWV, Hudziak JJ, Roza SJ, Steegers EAP, Hofman A, Verhulst FC, White TJH, Stricker BHC, Tiemeier H. Maternal Use of Selective Serotonin Reuptake Inhibitors, Fetal Growth, and Risk of Adverse Birth Outcomes. Archives of General Psychiatry. 2012;69(7):706-14.

24. El Marroun H, White TJ, van der Knaap NJ, Homberg JR, Fernández G, Schoemaker NK, Jaddoe VW, Hofman A, Verhulst FC, Hudziak JJ, Stricker BH, Tiemeier H. Prenatal exposure to selective serotonin reuptake inhibitors and social responsiveness symptoms of autism: population-based study of young children. Br J Psychiatry. 2014 Aug;205(2):95-102.

25. Steenweg-de Graaff J, Tiemeier H, Steegers-Theunissen RPM, Hofman A, Jaddoe VWV, Verhulst FC, Roza SJ. Maternal Dietary Patterns During Pregnancy and Child Internalising and Externalising Problems. The Generation R Study. Clinical Nutrition. 2014;33(1):115-21.

26. Steenweg-de Graaff J, Roza SJ, Steegers EA, Hofman A, Verhulst FC, Jaddoe VW, Tiemeier H. Maternal folate status in early pregnancy and child emotional and behavioral problems: the Generation R Study. Am J Clin Nutr. 2012 Jun;95(6):1413-21.

27. Steenweg-de Graaff J, Ghassabian A, Jaddoe VW, Tiemeier H, Roza SJ. Folate concentrations during pregnancy and autistic traits in the offspring. The Generation R Study. Eur J Public Health. 2014 [Jul 31 Epub ahead of print]

28. Ghassabian A, Steenweg-de Graaff J, Peeters RP, Ross HA, Jaddoe VW, Hofman A, Verhulst FC, White T, Tiemeier H. Maternal urinary iodine concentration in pregnancy and children's cognition: results from a population-based birth cohort in an iodine-sufficient area. BMJ Open. 2014 Jun 12;4(6):e005520.

29. Rosa SJ, Lier van PAC, Jaddoe VWV, Steegers EAP, Mol HA, Mackenbach JP, Verhulst FC, Tiemeier H. Intrauterine Growth and Infant Temperamental Difficulties: The Generation R Study. J Am Assoc Child Adol Psychiat 2008; 47:264-72.

30. van Batenburg-Eddes T, de Groot L, Steegers EA, Hofman A, Jaddoe VW, Verhulst FC, Tiemeier H. Fetal programming of infant neuromotor development. The Generation R Study. Pediatr Res 2010; 67(2):132-7

31. Mil NH van, Steegers-Theunissen RPM, Motazedi E, Jansen PW, Hofman A, Jaddoe VWV, Steegers EAP, Verhulst FC, Tiemeier H. Low and high Birth weight and the risk of child ADHD Symptoms. J Pediatrics (conditionally accepted).

32. Mil van NH, Steegers-Theunissen RPM, Bongers-Schokking JJ, Marroun HE, Ghassabian A, Hofman A, Jaddoe VWV, Visser TJ, Verhulst FC, De Rijke YB, Steegers EAP, Tiemeier H. Maternal Hypothyroxinemia During Pregnancy and Growth of the Fetal and Infant Head. Reproductive Sciences. 2012;19(12):1315-22.

33. Roman GC, Ghassabian A, Bongers-Schokking JJ, Jaddoe VW, Hofman A, de Rijke YB, Verhulst FC, Tiemeier H. Association of Gestational Maternal Hypothyroxinemia and Increased Autism Risk. Ann Neurol. 2013;74(5):733-42.

34. Ghassabian A, El Marroun H, Peeters RP, Jaddoe VW, Hofman A, Verhulst FC, Tiemeier H, White T. Downstream effects of maternal hypothyroxinemia in early pregnancy: nonverbal IQ and brain morphology in school-age children. J Clin Endocrinol Metab. 2014 Jul;99(7):2383-90.

35 Rijlaarsdam J, Tiemeier H, Hofman A, Jaddoe VW, Mackenbach JP, Verhulst FC, Stevens GW. Home environments of infants: relations with child development through age 3. Epidemiol Community Health. 2013 Jan;67(1):14-20

36. Rijlaarsdam J, Stevens GW, van der Ende J, Hofman A, Jaddoe VW, Mackenbach JP, Verhulst FC, Tiemeier H. Economic Disadvantage and Young Children's Emotional and Behavioral Problems: Mechanisms of Risk. J Abnorm Child Psychol. 2013 Jan;41(1):125-3

37. Jansen PW, Raat H, Mackenbach JP, Jaddoe VW, Hofman A, van Oort FV, Verhulst FC, Tiemeier H. National Origin and Behavioural Problems of Toddlers: The Role of Family Risk Factors and Maternal Immigration Characteristics. J Abnorm Child Psychol. 2010 Nov;38(8):1151-64

38. Rijlaarsdam J, Stevens GW, van der Ende J, Arends LR, Hofman A, Jaddoe VW, Mackenbach JP, Verhulst FC, Tiemeier H. A brief observational instrument for the assessment of infant home environment: development and psychometric testing. Int J Methods Psychiatr Res. 2012 Sep;21(3):195-204

39. Székely E, Tiemeier H, Jansen PW, Jaddoe VW, Hofman A, Verhulst FC, Herba CM. Maternal depressive symptoms are associated with low fearfulness in preschoolers. J Clin Child Adolesc Psychol. 2014;43(5):791-8.

40. Cents RA, Diamantopoulou S, Hudziak JJ, Jaddoe VW, Hofman A, Verhulst FC, Lambregtse-van den Berg MP, Tiemeier H. Trajectories of maternal depressive symptoms predict child problem behaviour: the Generation R study. Psychol Med. 2013 Jan;43(1):13-25.

41. Székely E, Lucassen N, Tiemeier H, Bakermans-Kranenburg MJ, Van Ijzendoorn MH, Kok R, Jaddoe VW, Hofman A, Verhulst FC, Herba CM. Maternal depressive symptoms and sensitivity are related to young children's facial expression recognition: the Generation R Study. Dev Psychopathol. 2014 May;26(2):333-45.

42. Lucassen N, Tharner A, Van Ijzendoorn MH, Bakermans-Kranenburg MJ, Volling BL, Verhulst FC, Lambregtse-Van den Berg MP, Tiemeier H. The association between paternal sensitivity and infant-father attachment security: a meta-analysis of three decades of research. J Fam Psychol. 2011 Dec;25(6):986-92.

43. Kok R, Linting M, Bakermans-Kranenburg MJ, van Ijzendoorn MH, Jaddoe VW, Hofman A, Verhulst FC, Tiemeier H. Maternal sensitivity and internalizing problems: evidence from two longitudinal studies in early childhood. Child Psychiatry Hum Dev. 2013 Dec;44(6):751-65

44. Kok R, Lucassen N, Bakermans-Kranenburg MJ, van IJzendoorn MH, Ghassabian A, Roza SJ, Govaert P, Jaddoe VW, Hofman A, Verhulst FC, Tiemeier H. Parenting, corpus callosum, and executive function in preschool children. Child Neuropsychol. 2014;20(5):583-606.

45. Rijlaarsdam J, Stevens GW, Jansen PW, Ringoot AP, Jaddoe VW, Hofman A, Ayer L, Verhulst FC, Hudziak JJ, Tiemeier H Maternal Childhood Maltreatment and Offspring Emotional and Behavioral Problems: Maternal and Paternal Mechanisms of Risk Transmission. Child Maltreat. 2014 Mar 18;19(2):67-78

46. Kok R, van IJzendoorn MH, Linting M, Bakermans-Kranenburg MJ, Tharner A, Luijk MP, Székely E, Jaddoe VW, Hofman A, Verhulst FC, Tiemeier H. Attachment insecurity predicts child active resistance to parental requests in a compliance task. Child Care Health Dev. 2013 Mar;39(2):277-87.

47. Tharner A, Dierckx B, Luijk MP, van Ijzendoorn MH, Bakermans-Kranenburg MJ, van Ginkel JR, Moll HA, Jaddoe VW, Hofman A, Hudziak JJ, Verhulst FC, Tiemeier H. Attachment disorganization moderates the effect of maternal postnatal depressive symptoms on infant autonomic functioning. Psychophysiology. 2013 Feb;50(2):195-203.

48. Sajjad A, Tharner A, Kiefte JC, Jaddoe VWV, Hofman A, Verhulst FC, Franco OH, Tiemeier H, Roza SJ.. Breastfeeding Duration and Non-verbal IQ in Children. J Epidemiol Commun Health (conditionally accepted)

49. Verlinden M, Tiemeier H, Hudziak JJ, Jaddoe VWV, Raat H, Guxens M, Hofman A, Verhulst FC, Jansen PW. Television Viewing and Externalizing Problems in Preschool Children: The Generation R Study. Archives of Pediatrics and Adolescent Medicine. 2012;166(10):919-25.

50. Verlinden M, Tiemeier H, Veenstra R, Mieloo CL, Jansen W, Jaddoe VW, Raat H, Hofman A, Verhulst FC, Jansen PW. Television viewing through ages 2-5 years and bullying involvement in early elementary school. BMC Public Health. 2014 Feb 12;14:157

51. Jansen PW, Verlinden M, Dommisse-van Berkel A, Mieloo CL, Raat H, Hofman A, Jaddoe VW, Verhulst FC, Jansen W, Tiemeier H. Teacher and peer reports of overweight and bullying among young primary school children. Pediatrics. 2014 Sep;134(3):473-80.

52. Verlinden M, Veenstra R, Ghassabian A, Jansen PW, Hofman A, Jaddoe VW, Verhulst FC, Tiemeier H. Executive functioning and non-verbal intelligence as predictors of bullying in early elementary school. J Abnorm Child Psychol. 2014 Aug;42(6):953-66.

53. Ghassabian A, Herba CM, Roza SJ, Govaert P, Schenk JJ, Jaddoe VW, Hofman A, White T, Verhulst FC, Tiemeier H. Infant Brain Structures, Executive Function, and Attention Deficit/Hyperactivity Problems at Preschool Age. A Prospective Study. Journal of Child Psychology and Psychiatry 2013;54(1):96-104.

54. Herba CM, Roza SJ, Govaert P, van Rossum J, Hofman A, Jaddoe V, Verhulst FC, Tiemeier H. Infant brain development and vulnerability to later internalizing difficulties: the Generation R Study. J Am Acad Child Adolesc Psychiatry. 2010;49:1053-63

55. Tharner A1, Herba CM, Luijk MP, van Ijzendoorn MH, Bakermans-Kranenburg MJ, Govaert PP, Roza SJ, Jaddoe VW, Hofman A, Verhulst FC, Tiemeier H Subcortical structures and the neurobiology of infant attachment disorganization: a longitudinal ultrasound imaging study. Soc Neurosci. 2011;6(4):336-47.

56. Mous SE, Muetzel RL, El Marroun H, Polderman TJ, van der Lugt A, Jaddoe VW, Hofman A, Verhulst FC, Tiemeier H, Posthuma D, White T.Cortical thickness and inattention/hyperactivity symptoms in young children: a population-based study. Psychol Med. 2014 Nov;44(15):3203-13.

57. Blanken LME, Mous SE, Ghassabian A, Muetzel RL, Schoemaker NK, El Marroun H, van der Lugt A, Jaddoe VWV, Hofman A, Verhulst FC, Tiemeier H, White T. Cortical morphology in 6-to-10 year old children with autistic traits – A population-based neuroimaging study. Am J Psychiat 2014, accepted for publication

58. Langeslag SJ, Schmidt M, Ghassabian A, Jaddoe VW, Hofman A, van der Lugt A, Verhulst FC, Tiemeier H, White TJ.Functional connectivity between parietal and frontal brain regions and intelligence in young children: the Generation R study. Hum Brain Mapp. 2013 Dec;34(12):3299-307.
